# Role of probiotics in preventing *Clostridioides difficile* infection in older adults: an integrative review

**DOI:** 10.3389/fmed.2023.1219225

**Published:** 2023-08-10

**Authors:** Maria Lucianny Lima Barbosa, Mariana Oliveira Albano, Conceição da Silva Martins, Cirle Alcantara Warren, Gerly Anne de Castro Brito

**Affiliations:** ^1^Morphology Department, Medical School, Federal University of Ceará, Fortaleza, Brazil; ^2^Division of Infectious Diseases and International Health, University of Virginia, Charlottesville, VA, United States

**Keywords:** *Clostridium difficile*, microbiota, diarrhea, infection, antibiotics, *Saccharomyces boulardii*, elderly

## Abstract

*Clostridioides difficile* infection (CDI) is the leading cause of healthcare-associated diarrhea. This infection can particularly affect older adults, the most susceptible to CDI. Currently, the standard therapeutic measure is antibiotic therapy, which in turn increases the risk of recurrence of the infection by its collateral damage to the patient’s microbiota. Probiotics are live microorganisms capable of maintaining balance in the intestinal microbiota. This study aims to perform an integrative review of the protective benefit of probiotics in CDI and diarrhea associated with *C. difficile*. The PubMed, Scopus, and Web of Science databases, the 10-year time cutoff, and the Prism Flow diagram were used for data collection. We observed no consensus among the studies; however, three of the seven evaluated studies demonstrated that the use of probiotics in older adults could contribute to reducing the incidence of hospital-onset CDI. We also found that the studies evaluated a wide variety of microorganisms, particularly *Saccharomyces boulardii*, associated with beneficial effects. More research is needed to understand the successful use of probiotics in the prevention of CDI in hospitalized older adults receiving antibiotics.

## 1. Introduction

*Clostridioides difficile* or *Clostridium difficile (C. difficile)* is a Gram-positive, spore-forming anaerobic bacillus that is abundantly distributed in the intestinal tract of humans and animals. *Clostridioides difficile* infection (CDI) is a significant cause of healthcare-associated diarrhea in many countries. It is associated with prolonged hospitalization and high mortality rates, thus placing a significant economic burden on healthcare systems. Transmission primarily occurs through the fecal-oral route, and its clinical manifestations range from asymptomatic carrier status to various degrees of diarrhea and even life-threatening colitis (1–3). Notably, *C. difficile*-associated diarrhea is most observed in patients receiving broad-spectrum antibiotics, residents of nursing homes, and in hospitalized older patients (4).

The disruption of the intestinal microbiota, particularly in older adults, has been identified as a significant risk factor for CDI, since the normal intestinal microbiota plays a crucial role in protecting against pathogenic bacteria. It is known that the balance of the intestinal microbiota of older adults can be disturbed, due to various factors, including the natural aging process and exposure to antibiotic therapy. Consequently, probiotics have emerged as a potential preventive and therapeutic strategy for managing gastrointestinal conditions, including diarrhea (5, 6).

Probiotics are live microorganisms that, when administered in adequate amounts, confer a health benefit to the host (7). In a meta-analysis by Lau; Chamberlain (8), probiotics were beneficial in reducing the risk of CDI in adults (RR = 0.405; 95% CI, 0.294–0.556; P, 0.001) and children (RR = 0.341; 95% CI, 0.153–0.759; *P* = 0.008). However, a study produced by Jafarnejad et al. (9) showed no significant beneficial effect of probiotic supplementation on antibiotic-associated diarrhea in older adults (>65 years), although another study shows contrary evidence in patients over 60 years old (10). A review study with a time cut from 1978 to 2015 evaluated the effectiveness of probiotics in reducing the incidence of *Clostridioides difficile*-associated diarrhea in elderly patients. It concluded that probiotics were not effective for the treatment. However, the authors recommend the execution of studies that evaluate new data since the data were scarce and heterogeneous (11). Thus, our study proposes to remedy this gap in the literature, considering more recent studies.

In view of the greater susceptibility of older people to CDI, and its adverse outcomes, as well as the inconsistencies in the literature regarding the beneficial effect of probiotics in preventing CDI in older adults, we conducted an integrative review of the current literature on the topic.

## 2. Materials and methods

Publications indexed in the Web of Science, PubMed and Scopus databases were analyzed. The search was limited to articles published between 2011 and 2021, available in English. The following combinations of English descriptors were used: (”Clostridium difficile infection”[Title]) OR (”Clostridium difficile” [Title]) OR (”Clostridioides difficile” [Title]) AND (”Diarrhea” [Title/Abstract]) OR (”Watery stools” [Title/Abstract]) AND (”Probiotics” [Title/Abstract]) OR (lactobacilli [Title/Abstract]) OR (Bifidobacterium [Title/Abstract]) AND (”Aged” [Title/Abstract]) OR (”Older adults” [Title/Abstract]) OR (”Elderly” [Title/Abstract]) NOT (”Fecal microbiota transplantation”[Title]). The study question was decided by the Patient, Intervention, Comparison and Outcome (PICO) strategy, where the study population was older adults, the intervention consisted of probiotic administration, and the outcomes (decreased incidence of CDI and diarrhea) were compared with subjects who did not receive probiotics or who received placebo.

Clinical and cohort trials that compared probiotic use with a concurrent or retrospective control group that received no treatment or only a placebo were included. The original articles addressed the effect of probiotic microorganism intervention in preventing CDI in older adults. Outcomes such as watery stools, stool consistency, self-reported diarrhea, and physician-defined diarrhea were included in this analysis. Articles that were not available in full were excluded, as well as review articles, dissertations, letters, opinions or perspectives, commentaries, reviews or books, and papers that evaluated other types of diarrhea than those associated with *C. difficile*.

The database search was conducted between September 2021 and January 2022. The CAPES Portal de Periódicos was used to obtain free, full-text articles.

## 3. Data extraction and analysis

The data were organized and categorized into a table in the Microsoft Excel 2010^®^ program, where the information regarding the identification of the publication, the research objectives, the type of study, and the level of evidence were organized.

A data extraction table that included information about the study population demographics, the sample size, the outcome variables, the probiotics used, and the dose administered was created.

Each article in the review was graded according to its level of evidence and assessed for study quality in the domains of sample selection, analysis of exposures and outcomes, and data analysis.

The selection, inclusion, and exclusion process of articles was presented using PRISMA flow (12).

## 4. Results and discussion

In the present study, we conducted a review of the current literature on the efficacy of the use of probiotics in CDI in older individuals. We observed that three of the seven evaluated studies demonstrated that using probiotics in the older population could decrease the incidence of CDI. We also found that the studies evaluated various strains; however, *Saccharomyces boulardii (S. boulardii)* was the one most associated with beneficial effects.

**FIGURE 1:**
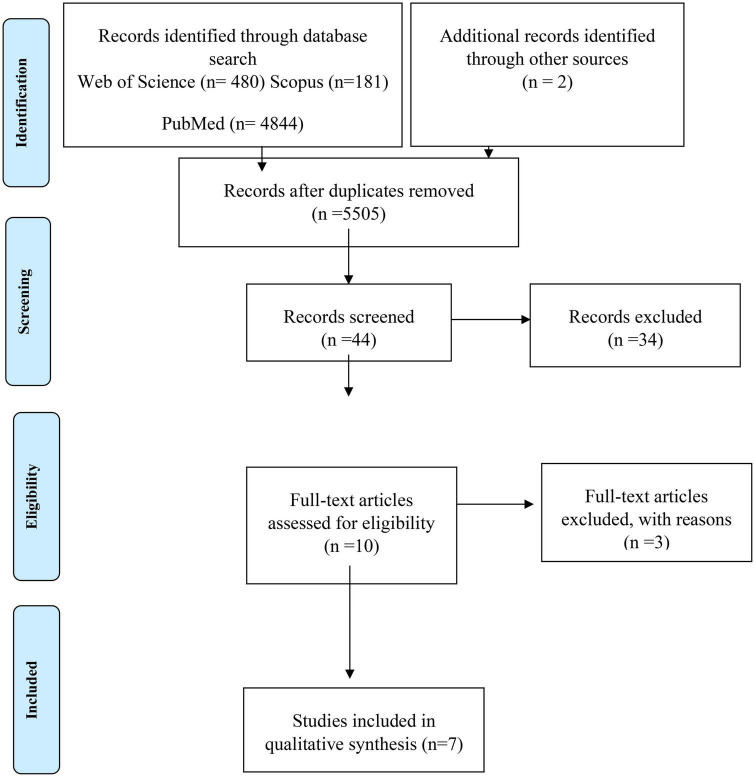
PRISMA flow diagram.

The details of the identification, selection, and exclusion process are shown in the PRISMA Flow diagram (Figure 1), and the baseline characteristics of the studies are described in Table 1. Only two of the studies involved more than one center (13, 14). The study population consisted of hospitalized patients, and the surveys were conducted in the United States (3), the United Kingdom (2), Denmark (1), and Japan (1). Among all the trials, the mean age range for patients assigned to the probiotic group was 74.8 years, and for placebo it was 74.6 years (*p* = 0.693).

**TABLE 1:** Studies that have evaluated the effect of using probiotics to prevent CDI in older patients receiving antibiotics.

Type of study	AGE	Population (*n*)	Probiotic/dose	Period	Main results	Quality	References
Multicenter, randomized, double-blind placebo-controlled, parallel arm trial (PLACIDE)	77.1 (71.0–83.5)	2,941	Lactobacilli and Bifid bacteria 6 ± 10^10^/day	21 days	Probiotic administration was not effective in reducing CDI in older patients exposed to antibiotics.	Low bias risk	Allen et al. (13)
Retrospective review	65.7 ± 18.4	17,119	*Saccharomyces boulardii* 250 mg two times/day	–	Administration of probiotics with antibiotics was not effective in preventing hospital-onset CDI.	Medium Bias Risk	Flatley et al. (16)
Retrospective cohort study	84 ± 4.37	238	*Lactobacillus casei*, *Lactobacillus bulgaricus*, and *Streptococcus thermophiles* (ACTIMEL) 100 gm (97 ml)	48 h after starting the antibiotics until 3 days after the last dose.	Probiotics were not effective in reducing the incidence of CDI in older patients receiving antibiotics	Medium Bias Risk	Mallina et al. (18)
Retrospective cohort study	75	43,379	*Saccharomyces boulardii* 5 ± 10^9^ (twice a day)	Use in combination with antibiotic treatment for at least 7 days after discharge	There was a significant reduction in the incidence of antibiotic-induced CDI in a hospital population receiving prophylactic probiotic capsules	Medium Bias Risk	Carstensen et al. (18)
Retrospective cohort study	65.8 ± 18.7	1,576	*Lactobacillus acidophilus* CL1285, *Lactobacillus casei* LBC80R, and *Lactobacillus rhamnosus* CLR2	8 days	There was no difference in the rates of HO-CDI between hospitalized patients who received antibiotics, with or without probiotics.	High Bias Risk	Box et al. (17)
Retrospective case-control study	83.3	149	*Streptococcus faecalis* (2 ± 10^8^ CFU/day),Bacillus mesentericus (1 ± 10^7^ CFU/day), and *Clostridium butyricum* (5 ± 10^7^ CFU/day).	14 days	Treatment of older patients with a combination of probiotics after undergoing orthopedic surgery reduced the likelihood of CDI;	Medium Bias Risk	Nagamine et al. (19)
Retrospective cohort study	64.3 ± 18.4	8,763	*Saccharomyces boulardii* 1 ± 10^10^ (twice a day)	–	The combination of probiotic with antibiotic therapy often associated with HO-CDI in an older hospitalized patient population significantly reduced the incidence of HO-CDI.	Low bias risk	Wombwell et al. (15)

The probiotic *S. boulardii* was investigated in three studies. Carstensen et al. (14) showed a significant reduction in the incidence of antibiotic-induced CDI in the population that received *S. boulardii*, once a day, concomitantly with antibiotic treatment. Wombwell et al. (15) demonstrated that co-administration of probiotic *S. boulardii* with antibiotic therapy in an older hospitalized patient population significantly reduced the incidence of hospital-onset CDI (HO-CDI) (16). On the other hand, Flatley Wilde, and Nailor (16) showed that the removal of a routine instruction to initiate *S. boulardii* therapy to patients receiving antibiotics did not impact the rate of CDI. Another three studies evaluated the use of a mix of probiotic-containing species of the genus *Lactobacillus* combined (17), or associated with *Streptococcus thermophiles*(18), or with species of the genus Bifidobacteria (13). Box et al. (17) observed no impact of probiotics containing a combination of species of *Lactobacillus* in reducing the incidence of CDI. Mallina et al. (18) also demonstrated no significant reduction in the incidence of CDI in patients receiving *Lactobacillus* associated with *Streptococcus thermophiles*. In the same way, Allen et al.(13) showed that the administration of *Lactobacillus* associated with species of the genus Bifidobacteria did not prevent CDI. On the other hand, Nagamine et al. (19) indicated that a combination of *Streptococcus faecalis*, *Bacillus mesentericus*, and *Clostridium butyricum* successfully prevented CDI. Table 1 presents a summary of the main results of the selected studies and of the demographics data.

Regarding the methodological quality of the selected articles, it was found that all articles demonstrated which comparison was being made. However, except for the study by Allen et al. (13), the studies were not randomized clinical trials, which could lead to potential selection biases and confounding factors. Despite the methodological weakness typical of retrospective studies, it was observed that some authors made a systematic effort to identify, measure, and mitigate potential selection biases.

The most robust study in this review (13) showed no significant effect of probiotic intervention in reducing antibiotic-induced diarrhea. However, it should be noted that this study showed a low percentage of risk for developing CDI (1.2%). A systematic review (20) found that the baseline risk of developing CDI may influence intervention effectiveness with probiotics in adults and children. That study showed that only trials with a baseline risk ≥5% showed a statistically significant treatment effect.

It is worth mentioning that the PLACIDE study protocol (13) specified initiation of the probiotic within 7 days of starting antibiotics. However, the systematic review by Shen et al. (21) showed that studies that required early administration of probiotics found significantly higher efficacy, so probiotics administered within 2 days after the first dose of antibiotic were more effective than the late-onset protocols. Despite these considerations, the most recent American College of Gastroenterology guideline included the PLACIDE study as one of the most important studies examining the efficacy of probiotics in CDI (22). *S. boulardii*, a probiotic strain, can produce a protease that inactivates the receptor site for *C. difficile* toxin A, conferring biological plausibility to its use in CDI (23). Three of the studies presented in this review investigated the effect of the *S. boulardii* strain on the incidence of hospital-onset CDI (14–16). The study by Flatley, Wilde, and Nailor (16), in turn, presented a different methodology since the main objective of the study was to evaluate the effect on the incidence of CDI-HO of removing the *S. boulardii* strain from an automated hospital antibiotic request form. The authors found that the incidence during and after protocol removal was 1.25 vs. 1.51% (*p* = 0.70). Limitations of the study include the fact that it is a retrospective review and, therefore, the lack of effect cannot be explicitly linked to using the strain or measured in the control group.

The study by Carstensen et al. (14) investigated the incidence of CDI in four hospitals; in one of these hospitals patients who received broad-spectrum antibiotics also received *S. boulardii*. The CDI rate decreased from 3.6 to 1.5% in the hospital that received the probiotic. However, one of the control hospitals also showed a significant reduction in the incidence of CDI; the authors attributed this reduction to adopting a multifaceted CDI intervention and introducing rigorous decontamination policies in this hospital. Among the limitations of the study is the inability to prove a causal relationship between *S. boulardii* and the observed risk reduction. However, a systematic review suggested that there may be some benefits to the use of this probiotic in the treatment and secondary (recurrence) prevention in adult patients presenting with CDI (24).

The work of Wombwell et al. (15) observed that co-administration of the probiotic *S. boulardii* at a dose of 20 billion CFUs per day with antibiotic therapy reduced the incidence of CDI. Interestingly, patients with *S. boulardii* co-administered within 24 h of antibiotic initiation demonstrated a lower risk of hospital-onset CDI than those co-administered after 24 h. This result corroborates the study by Shen et al., (21) using a meta-regression which states that a relationship exists between the time of initiation of probiotic co-administration and the outcome of whether the intervention was effective.

In a separate study, researchers investigated the effects of administering the probiotic *S. boulardii*, combined with the antibiotic amoxicillin-clavulanate on the microbiota and symptoms of healthy humans. That study revealed that when the probiotic was given alongside the antibiotic, it reduced changes in the microbiota (dysbiosis) and also alleviated antibiotic-associated diarrhea. These beneficial effects of the probiotic-antibiotic combination on the microbiota suggest that it could potentially offer protection against CDI (25).

Box; Ortwine, Goicoechea (17), in turn, observed no difference in hospital-onset CDI rates between patients receiving antibiotics, with or without probiotics. The authors investigated a combination of species from the genus Lactobacillus. Limitations of the study include the inability to determine the timing of probiotics concerning the first dose of antibiotics; in addition, probiotics were administered using a “standard dose” that was not defined.

In another study, Mallina et al. (18) evaluated the use of probiotics to prevent CDI in a group of high-risk orthogeriatric patients. They found that the probiotic drink ACTIMEL was ineffective in reducing CDI in older adults. A systematic review by the Cochrane group found that probiotics were effective in reducing CDI only in children and adults (20). Older adults are part of the group most vulnerable to the effects of antibiotics, which may occur due to the association between aging and changes in the microbiota (26), making it more challenging to restore.

Nagamine and colleagues (19) investigated the effect of a probiotic combination consisting of *Streptococcus faecalis*, *Clostridium butyricum*, and *Bacillus mesentericus*. They suggested that these probiotics had a role in the primary prevention of CDI. A potential mechanism of action of the antimicrobial activities of probiotics includes the production of bacteriocins/defensins, competitive inhibition with pathogenic bacteria, inhibition of bacterial adhesion or translocation, reduction of luminal pH, and increased mucus production that would also contribute to improved intestinal barrier function (27).

**FIGURE 2:**
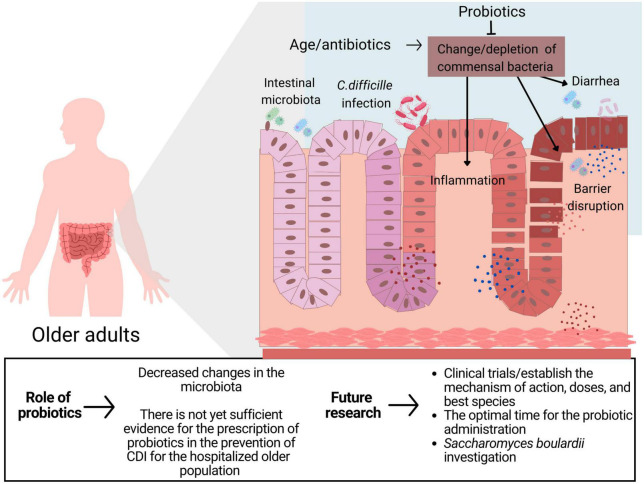
The role of probiotics in CDI in older adults.

The present work has many limitations, as it is a review of mostly retrospective cohort studies, which may confer a margin of error for possible selection biases. However, it is worth noting that the results of clinical trials may not apply to the general population due to strict eligibility criteria. Real-world studies, on the other hand, complement clinical trials by generalizing the clinical trial findings to the general population (28). Another limitation of the present study is the wide variety of genera, species, and probiotic doses included, which may have imparted heterogeneity to the study. However, our choice not to limit the research to only one genus or species is guided by Goldenberg et al. (20) using the hypothesis that there is a similarity in the mechanism of action of different types of probiotics and that any variation in effect would be due to chance. Figure 2 presents the main results of this study as well as suggestions for further research.

The clinical implication of the present study is that although some papers demonstrate the efficacy of probiotic use, there is not yet sufficient evidence to support the prescription of probiotics in older adults to prevent CDI. This recommendation is in line with the most recent guidelines published by the Society for Healthcare Epidemiology of America and the American College of Gastroenterology (22). Interestingly, *S. boulardii* species appeared to be most associated with beneficial effects in the prevention of CDI. This finding is consistent with the American Gastroenterological Association (AGA) 2020 guidelines that recommended the use of specific strains and combinations of strains, including *S. boulardii*, to prevent CDI in adults and children on antibiotic treatment (29). This recommendation, however, still has a low level of evidence and should only be extrapolated to the older population in a clinical trial setting. More clinical trials are needed that specifically target the treatment and prevention of CDI in older patients. Clinical studies with standardized protocols that determine the optimal time for the initiation of probiotic administration, identify specific probiotics that are beneficial and which populations are most suitable for their use and take into consideration other factors, such as diet, that significantly interfere with the human microbiota, would be most relevant. In addition, cost-benefit analyses of the use of probiotics for this purpose are valuable.

## 5. Conclusion

There is not yet sufficient evidence for the prescription of probiotics in the prevention of CDI for the hospitalized older population taking antibiotics. Robust clinical studies that include the *S. boulardii* strain are needed to address the scientific and clinical gaps on the potential protective effect of probiotics on CDI.

## Author contributions

MB, CM, and GB developed the study concept. MB and MA drafted the manuscript. CW and GB revised the critically manuscript. MB developed the search strategy. All authors have read and agreed to the published version of the manuscript.
